# Tumor tissue protein signatures reflect histological grade of breast cancer

**DOI:** 10.1371/journal.pone.0179775

**Published:** 2017-06-26

**Authors:** Petter Skoog, Mattias Ohlsson, Mårten Fernö, Lisa Rydén, Carl A. K. Borrebaeck, Christer Wingren

**Affiliations:** 1 Dept. of Immunotechnology, Lund University, Medicon Village, Lund, Sweden; 2 Computational Biology & Biological Physics, Department of Astronomy and Theoretical Physics, Lund University, Lund, Sweden; 3 Department of Oncology and Pathology, Clinical Sciences, Lund University, Lund, Sweden; 4 Department of Surgery, Clinical Sciences, Lund University, Lund, Sweden; University of South Alabama Mitchell Cancer Institute, UNITED STATES

## Abstract

Histological grade is one of the most commonly used prognostic factors for patients diagnosed with breast cancer. However, conventional grading has proven technically challenging, and up to 60% of the tumors are classified as histological grade 2, which represents a heterogeneous cohort less informative for clinical decision making. In an attempt to study and extend the molecular puzzle of histologically graded breast cancer, we have in this pilot project searched for additional protein biomarkers in a new space of the proteome. To this end, we have for the first time performed protein expression profiling of breast cancer tumor tissue, using recombinant antibody microarrays, targeting mainly immunoregulatory proteins. Thus, we have explored the immune system as a disease-specific sensor (clinical immunoproteomics). Uniquely, the results showed that several biologically relevant proteins reflecting histological grade could be delineated. In more detail, the tentative biomarker panels could be used to i) build a candidate model classifying grade 1 vs. grade 3 tumors, ii) demonstrate the molecular heterogeneity among grade 2 tumors, and iii) potentially re-classify several of the grade 2 tumors to more like grade 1 or grade 3 tumors. This could, in the long-term run, lead to improved prognosis, by which the patients could benefit from improved tailored care.

## Introduction

More women are diagnosed with breast cancer than any other cancer form, affecting one in eight women during their lives [1] [[https://seer.cancer.gov/]. For patients diagnosed with breast cancer, histological grade is one of the most commonly used prognostic factors [2, 3]. Histological grade describes the aggressive potential of the tumor, and is a combined score based on microscopic evaluation of the tubule formation, mitotic count, and nuclear pleamorphism [2, 3]. While grade 3 tumors are the most aggressive (highly proliferative) and poorly differentiated, grade 2 tumors are moderately differentiated, and grade 1 tumors are the least aggressive (slow growing) and well-differentiated [3].

However, concerns have been raised regarding the prognostic value of histological grade [4, 5]. These concerns reflect the shortcomings associated with conventional grading of breast cancer tumors using methods based on visual evaluation [4–8]. The patient cohort with grade 2 classified tumors, representing 30 to 60% of all patients, are in particular difficult to manage, as these tumors are very heterogeneous and less informative for clinical decision making [9]. Hence, new improved means to perform histological grading of breast cancer, and especially grade of grade 2 tumors, would thus be of significant clinical value.

Several attempts have been made to subgroup breast cancer based on predominantly genetic signatures (*e*.*g*. reflecting prognosis and treatment outcome) [10–14], but also proteomic biomarker signatures [15–21]. Noteworthy, the genetic biomarker signatures have not only highlighted the heterogeneity of grade 2 tumors, but also indicated that it might be possible to re-classify this cohort into one subgroup more similar two grade 1 tumors and one subgroup more similar to grade 3 tumors [9, 12]. The potential subdivision of grade 2 tumors have also been indicated targeting the proliferation marker Ki-67 using immunohistochemistry [8, 22]. Despite the success, additional high-performing biomarkers must be deciphered in order to pave the way for grading of breast cancer tumors based on molecular portraits.

Using a mass spectrometry-based affinity approach [23], we have deciphered a 49-plex candidate protein signature discriminating between histological grade 1, 2, and 3 classified breast cancer [24]. These findings have recently been extended and further refined using a targeted mass spectrometry approach (Olsson et al, submitted). While grade 1 tumors were found to express higher levels of extra cellular matrix associated proteins and stromal proteins, indicating a more conserved structure, grade 3 tumors expressed higher levels of proteins associated to proliferation and mitosis, at the same time losing the structural properties. Notably, the grade 2 tumors were found to be heterogeneous, indicating that many of them could be re-classified as being more similar to grade 1 or grade 3 tumors, respectively.

In an attempt to further study and extend this molecular puzzle of histologically graded breast cancer, we have in this pilot project searched for additional protein biomarkers in a new space of the proteome. To this end, we have for the first time performed protein expression profiling of breast cancer tissue, using recombinant antibody microarrays, targeting mainly immunoregulatory proteins [25–28]. Thus, we have explored the immune system as a disease-specific sensor (clinical immunoproteomics) [29] to reflect histological grade of breast cancer. The results showed that novel candidate biomarker signatures, based on immunoregulatory proteins, reflecting histological grade of breast cancer, and in particular the heterogeneity of grade 2 tumors, could be delineated, adding new key pieces to the underlying molecular puzzle.

## Material and methods

### Clinical samples

This study was approved by the regional ethics board at Lund University, Sweden (LU240-01). Fifty primary breast cancer patients were recruited from the South Sweden Breast Cancer Groups tumor bank (Lund, Sweden). Freshly frozen breast tumor tissues were stored at -80°C until analysis. Full clinical records, including tumor size, steroid receptor status [30], and lymph node involvement were at hand (Table 1). The breast tumor samples were subdivided based on Nottingham histological grades 1 (n = 9), 2 (n = 17), and 3 (n = 24), by trained pathologists at the Department of Pathology (Skane University Hospital).

**Table 1:** Patient demographics and clinical parameters.

Parameter	Histological grade 1	Histological grade 2	Histological grade 3
Number of patients	9	17	24
Age in years	55.8 (11.9)^a^	45.9 (4.0)	45.8 (5.2)
Tumor size [mm]	24.3 (5.9)	21.9 (10.9)	29.5 (9.2)
ER+/ER-^b^	9/0	14/3	10/14
PgR+/PgR-	9/0	13/4	11/13
Lymph node+ / Lymph node -	5/4	14/3	14/10
Her2+ / Her2-^c^	0/9	0/16^§^^d^	5/15
Ki67+/Ki67-	0/8	4/9	14/6

^a)^ Values in parenthesis is standard deviation

^b)^ Estrogen Receptor status (ER) and Progesterone Receptor status (PgR) were analyzed in cytosol samples with ligand binding assays (LBA) or enzyme immunoassay (EIA) as previously described^11^. Samples with receptor content higher or equal to 10 (LBA) or 25 (EIA) fmol/mg protein were classified as ER or PgR positive, and samples with values below these levels as ER or PgR negative

^c)^ All patients with FISH (fluorescence *in situ* hybridization) amplified tumors and all patients with an immunohistochemical 3+, where FISH analysis could not be evaluated, were considered HER2+

^d)^ In cases where the sum is less than the number in the group, patient data are missing

### Extraction of proteins from solid tumors

Protein was extracted from solid breast cancer tumor tissue and stored at -80°C until use. Briefly, tissue pieces (about 50 mg/sample) were homogenized in Teflon containers with a metal ball, pre-cooled in liquid nitrogen, fixating the bomb in a shaker for two 30s periods with quick cooling in liquid nitrogen between the rounds. The homogenized samples were weighted and transferred to a collection tube and stored at -80°C until use. Extraction of proteins from homogenized tissue followed previously described protocol [31, 32]. Briefly, 10 μl of Extraction Buffer (100μg/ml Soybean trypsin inhibitor, 350 μg/ml PMSF, 0.01% (w/v) BSA and 2% (w/v) Saponin) was added per mg of sample and incubated on a rocking table at 4°C o/n. Samples were centrifuged at 13,000g for 5 minutes, and supernatant was transferred to a new tube and stored at -80°C until use.

Sample extracts were thawed on ice, and buffer was changed to PBS using Zeba desalt spin columns (Pierce Rockford, IL, USA). Protein concertation was determined using Total Protein Kit, Micro Lowry (Sigma-Aldrich, St. Louis, MO, USA). Biotinylation of samples was done using EZ-link Sulfo-NHS-LC Biotin (Pierce Rockford) according to a previously optimized protocol [26, 27, 32]. Briefly, samples were diluted to approximately 2 mg/ml and biotin was added at a molar ratio of protein:biotin of 1:15, with a final biotin concentration of 0.6 mM. Reaction between biotin and protein was done at 4°C for 2 hours, and excess biotin was removed through dialysis against PBS for 72 hours at 4°C. Biotinylated samples were aliquoted and transferred to new tubes and stored at -20°C until use.

### Antibodies

In total, 293 human recombinant single-chain fragment variable (scFv) antibodies (Table 2) were selected from large phage display libraries [33, 34]. Out of 293 antibodies, 262 were targeted against 98 known serum antigens, reflecting mainly immunoregulatory proteins. Each target had 1–9 clones directed against it to ensure antibody reactivity, even if the epitope for one antibody clone was masked by the biotinylation. The remaining 31 antibodies were directed against short peptide motifs, 4–6 amino acids in length (antibodies are denoted as CIMS1-31) [35]. The scFv antibodies have previously been demonstrated to provide high on-chip funcationality [27, 36]. The specificity for several of the antibodies have been validated and tested in ELISA, mass spectrometry, Meso Scale Discovery assay and spiking and/or blocking experiments using standardized serum samples with known levels of the targeted analytes [26, 27, 31, 37–39].

**Table 2:** Antigens and number of clones against each.

Antibody clone (no)	Full name	Clone (no)	Full name
Angiomotin (1–2)	Angiomotin	IL-8 (1–3)	Interleukin-8
Apo-A1 (1–3)	Apolipoprotein A1	IL-9 (1–3)	Interleukin-9
Apo-A4 (1–3)	Apolipoprotein A4	Integrin α10 (1)	Integrin alpha-10
ATP-5B (1–3)	ATP synthase subunit β, mitochondrial	Integrin α11 (1)	Integrin alpha-11
B-galactosidase (1)	Beta-galactosidase	JAK3 (1)	Tyrosine-protein kinase JAK3
BTK (1–4)	Tyrosine-protein kinase BTK	Keratin 19 (1–3)	Keratin, type I cytoskeletal 19
C1 inh. (1–4)	Plasma protease C1 inhibitor	KSYK (1–2)	Tyrosine-protein kinase SYK
C1q (1)	Complement C1q	LDL (1–2)	Apolipoprotein B-100
C1s (1)	Complement C1s	Leptin (1)	Leptin
C3 (1–6)	Complement C3	Lewis x (1–2)	Lewis x
C4 (1–4)	Complement C4	Lewis y (1)	Lewis y
C5 (1–3)	Complement C5	LUM (1)	Lumican
CD40 (1–4)	CD40 protein	MAPK1 (1–4)	Mitogen-activated protein kinase 1
CD40 ligand (1)	CD40 ligand	MAPK8 (1–3)	Mitogen-activated protein kinase 8
CDK-2 (1–2)	Cyclin-dependent kinase 2	MATK (1–3)	Megakaryocyte-associated tyrosine-protein kinase
CHX10 (1–3)	Visual system homeobox 2	MCP-1 (1–9)	C-C motif chemokine 2
CIMS (1–31)		MCP-3 (1–3)	C-C motif chemokine 7
CT17 (1)	Cholera Toxin subunit B	MCP-4 (1–3)	C-C motif chemokine 13
Cystatin C (1–4)	Cystatin-C	MUC1 (1–6)	Mucin-1
Digoxin (1)	Digoxin	MYOM2 (1–2)	Myomesin-2
DUSP9 (1)	Dual specificity protein phosphatase 9	ORP-3 (1–2)	Oxysterol-binding protein-related protein 3
Eotaxin (1–3)	Eotaxin	Osteopontin (1–3)	Osteopontin
Factor B (1–4)	Complement factor B	P85A (1–3)	Phosphatidylinositol 3-kinase regulatory subunit α
FASN (1–4)	FASN protein	PKB gamma (1–2)	RAC-gamma serine/threonine-protein kinase
GAK (1–3)	GAK protein	Procathepsin W (1)	Cathepsin W
GLP-1 (1)	Glucagon-like peptide-1	Properdin (1)	Properdin
GLP-1 R (1)	Glucagon-like peptide 1 receptor	PSA (1)	Prostate-specific antigen
GM-CSF (1–6)	Granulocyte-macrophage colony-stimulating factor	PTK6 (1)	Protein-tyrosine kinase 6
HADH2 (1–4)	HADH2 protein	PTPN1 (1–3)	Tyrosine-protein phosphatase non-receptor type 1
Her2/ErbB2 (1–4)	Receptor tyrosine-protein kinase erbB-2	RANTES (1–3)	C-C motif chemokine 5
HLA-DR/DP (1)	HLA-DR/DP	RPS6KA2 (1–3)	Ribosomal protein S6 kinase α-2
ICAM-1 (1)	Intercellular adhesion molecule 1	Sialle x (1)	Siallelewis x
IFN-γ (1–3)	Interferon gamma	Sox11a (1)	Transcription factor SOX-11
IgM (1–5)	ImmunoGlobulin M	STAP2 (1–4)	Signal-transducing adaptor protein 2
IL-10 (1–3)	Interleukin-10	STAT1 (1–2)	Signal transducer and activator of transcription 1-α/β
IL-11 (1–3)	Interleukin-11	Surface Ag X (1)	Unknown surface antigen
IL-12 (1–4)	Interleukin-12	TBC1D9 (1–3)	TBC1 domain family member 9
IL-13 (1–3)	Interleukin-13	TENS4 (1)	Tensin-4
IL-16 (1–3)	Interleukin-16	TGF-b1 (1–3)	Transforming growth factor beta-1
IL-18 (1–3)	Interleukin-18	TM peptide (1)	Transmembrane peptide
IL-1a (1–3)	Interleukin-1 α	TNF-a (1–3)	Tumor necrosis factor
IL-1b (1–3)	Interleukin-1 β	TNF-b (1–4)	Lymphotoxin-alpha
IL-1ra (1–3)	Interleukin-1 receptor antagonist protein	TNFRSF14 (1–2)	Tumor necrosis factor receptor superfamily member 14
IL-2 (1–3)	Interleukin-2	TNFRSF3 (1–3)	Tumor necrosis factor receptor superfamily member 3
IL-3 (1–3)	Interleukin-3	UBC9 (1–3)	SUMO-conjugating enzyme UBC9
IL-4 (1–4)	Interleukin-4	UBE2C (1–2)	Ubiquitin-conjugating enzyme E2 C
IL-5 (1–3)	Interleukin-5	UCHL5 (1)	Ubiquitin carboxyl-terminal hydrolase isozyme L5
IL-6 (1–8)	Interleukin-6	UPF3B (1–2)	Regulator of nonsense transcripts 3B
IL-7 (1–2)	Interleukin-7	VEGF (1–4)	Vascular endothelial growth factor

All scFv antibodies were produced in 15 mL *E*. *coli* cultures and purified from periplasm, using MagneHis^™^ Protein Purification system (Promega, Madison, WI, USA) and a KingFisher96 robot (Thermo Fisher Scientific, Waltham, MA, USA). Elution Buffer from purification was exchanged for PBS using Zeba 96-well desalt spin plates (Pierce). Protein concentration for all antibodies were determined at 280 nm, using a NanoDrop-1000 (Thermo Scientific, Wilmington, DE, USA), and purity using 10% SDS-PAGE (Invitrogen, Carlsbad, CA, USA).

### Antibody microarrays

The antibody microarrays consisted of 293 scFv antibodies, printed on black polymer MaxiSorp slides (NUNC, Roskilde, Denmark) using a non-contact printer (SciFlexarrayer S11, Scienon, Berlin, Germany). Each slide was made up of 12 identical subarrays, each subarray containing three identical segments, divided by rows of biotinylated BSA. Intermixed with the antibodies, several negative controls (PBS) were spotted. In total, each subarray constituted of 31x33 spots. The slides were printed o/n, and subsequently used for analysis the following day.

Each slide was mounted in a hybridization gasket (Schott, Jena, Germany), creating individual well over each array. The array surface was blocked using PBSMT (1% (w/v) milk, 1% (v/v) Tween-20 in PBS) for one hour at room temperature (RT). During blocking, samples were thawed on ice and diluted 1:10 in PBSMT. Next, the slides were washed four times using PBST (1% (v/v) Tween-20 in PBS), followed by addition of 100 μl of sample, and subsequently incubated for 2 hours at RT on a rocking table. The slides were washed four times with PBST, and incubated with 1 μg/mL Streptavidin-Alexafluor647 (Invitrogen, Carlsbad, CA, USA) in dark conditions on a rocking table for one hour at RT, and subsequently washed four times using PBST. Next, the slides were dismounted from the gaskets, and slowly dipped in dH_2_O where after slides were directly dried under a stream of nitrogen gas. Thereafter, the slides were immediately scanned in a confocal scanner (PerkinElmer Life and Analytical Sciences, Wellesley, MA, USA) at 10 μm resolution, using 60% PMT gain and 90% laser power. The resulting signal intensities were quantified using ScanArray Express Software version 4.0 (Perkin Elmer Life and Analytical Sciences), using the fixed circle method. Intensity values with local background subtraction were used in the subsequent data analysis steps.

### Data preprocessing

The mean value was calculated for each target protein using the three scFv replicate spots. In those cases, where one replicate CV deviated more than 15%, the mean of the two remaining signals was used. The average replicate CV was 7.2% (±5.5%). Setting the cut-off value for CV at 15%, 81.7% of data points were obtained using three replicates, and the remaining data points was obtained using the remaining two replicates.

Next, three scFv were discarded as their mean signal intensities were below the limit of detection (LOD) cut-off (defined as mean_PBS_+2(SD_PBS_) in >70% of the samples), leaving 290 scFvs in the dataset.

Using QluCore Omics Explorer 3.0 (Qlucore, Lund, Sweden), a 3D principle component analysis (PCA) plot on log2 raw data, an initial exploration of the dataset was performed. This approach was combined by visual inspection of microarray digital images. As a result, one sample was removed from the data analysis, since it was regarded as an outlier in the PCA plot. With the PCA plot, it could also be determined that neither position on the slide, hormonal status, nor grading had introduced any observable difference or influence.

Next, the array-to-array variations were handled by using a semiglobal normalization approach [25, 38, 40, 41]. Thus, the coefficient of variation (CV) was calculated for each analyte and ranked. Fifteen percent of the scFvs displaying the lowest CV values over all samples were identified, corresponding to 44 analytes. These analytes were used to calculate a normalizing factor [42]. The normalization factor, N_i_, for each sample i, was calculated by the formula N_i_ = S_i_/μ, where S_i_ is the sum of the signal intensities for the 44 analytes for each sample i and μ is the average of all S_i_. Each dataset generated from one sample was divided with the normalization N_i_. For the intensities, log2 values were used in the analysis.

### Data analysis

All statistics and data analysis was performed in the program R (www.r-project.com) [43]. The Support Vector Machine (SVM) is a supervised learning method in R [43–45] used to classify the samples. The supervised classification was performed using a linear kernel, and the cost of constraint set to 1 to avoid overfitting. The SVM was trained using a leave-one-out cross-validation procedure (LOOC SVM). Briefly, training sets were created excluding one sample. The SVM was asked to classify the excluded sample as belonging to either group, and assign a decision value, *i*.*e*. the distance from a hyperplane. Unfiltered data, i.e. all analytes, were used in the process. This process was iterated for all samples, and a receiving operator characteristic (ROC) curve was constructed using the decision values, and the area under the curve (AUC) was calculated. Significantly up- or down-regulated proteins (p < 0.05) were identified using Wilcoxon signed-rank test.

In an attempt to further stratify samples from grade 2 into two groups, one more similar to grade 1 samples, the other more similar to grade 3, the dataset was divided into a training set with only grade 1 and 3 tumors, and a test set with only grade 2 tumors. A SVM-based Backward Elimination algorithm previously described described [41] was applied using the training set. The Kullback-Leibler (K-L) error in the classification was plotted against the number of eliminated antibodies. Based on the K-L error, a signature was defined as the 20 last remaining antibodies, and a classification model was built using the training set, and was then applied on the test.

To test grade 1 or grade 3 samples, it was imperative to exclude the samples from the training set. To achieve this objective, a bootstrap strategy was developed. Briefly, training sets were created with randomly drawn grade 1 or 3 samples, with resampling. Undrawn samples were added to the test set to be tested. Backward Elimination was run for each training set, and a classification model was built for each iteration and applied to the test set.

The raw array dataset is available as supporting information (S1 Table).

### Biomarker validation using an orthogonal method

A commercial ELISA kit against IL-6 (IL-6 ELISA Kit, Human, Cat#EH2IL6, Thermo Fisher Scientific) was run as an orthogonal method to validate the microarray results. The analysis used reagents supplied with the kit, and followed the protocol provided by the supplier. The absorbance was measured at 450 nm using a FLUOstar Omega plate reader (BMG LABTECH GmbH, Ortenburg, Germany). Raw data was used, and statistical analysis was performed using Welsh’s t-test in R to consider the unequal sample sizes.

## Results

In this study, we have performed tissue protein profiling of breast cancer tumors in an attempt to decode novel biomarkers reflecting histological grade. To this end, a 293-plex recombinant antibody microarray platform targeting the immunoproteome was used. Fifty breast cancer tumors, distributed between histological grade 1 (n = 9), 2 (n = 17), and 3 (n = 24) were profiled.

### Molecular grading of breast cancer tumors

**Fig 1:**
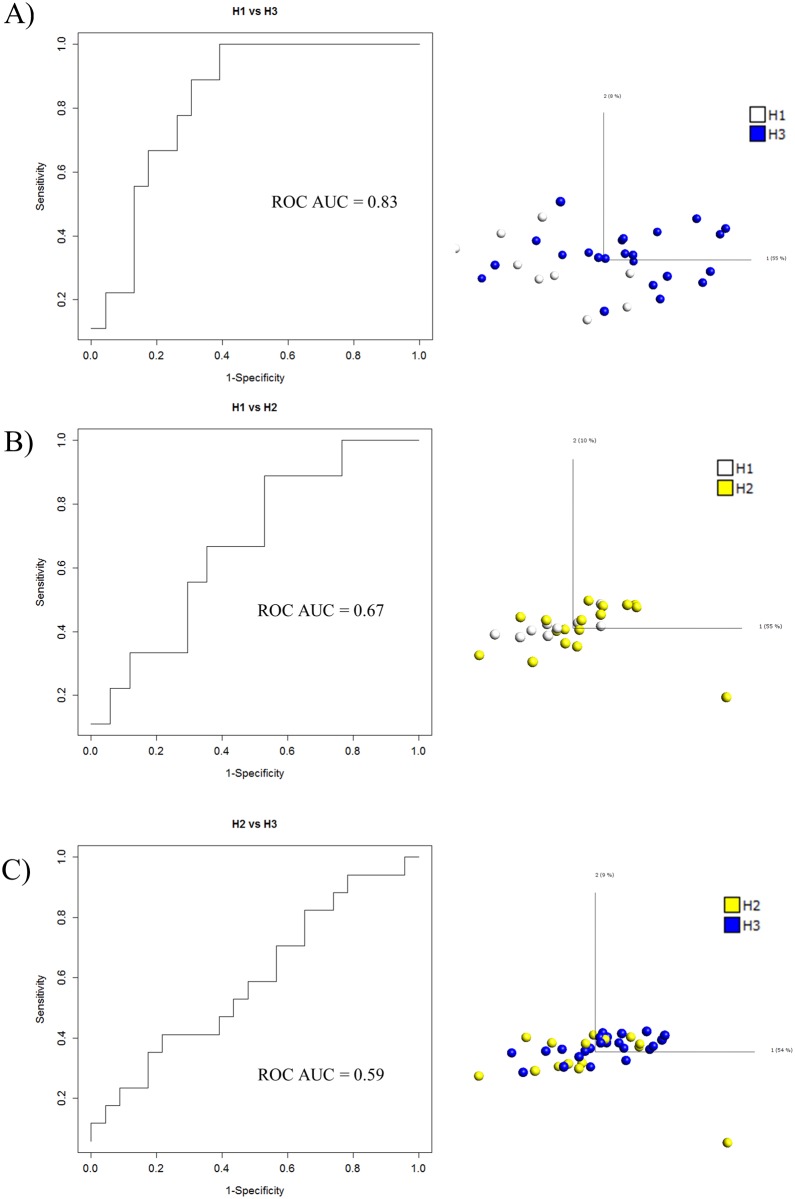
Molecular classification of breast cancer tumors according to histological grade (H1, H2, and H3) by tumor tissue protein expression profiling, using recombinant scFv antibody microarrays. Unfiltered data was used in all analysis. A) A ROC curve and AUC value obtained for H1 vs. H3, using a LOOC SVM (left panel). A PCA plot for H1 vs. H3 (right panel). B) A ROC curve and AUC value obtained for H1 vs. H2, using a LOOC SVM (left panel). A PCA plot for H1 vs. H2 (right panel). C) A ROC curve and AUC value obtained for H1 vs. H3, using a LOOC SVM (left panel). A PCA plot for H2 vs. H3 (right panel).

First, we explored whether tissue protein signatures classifying breast cancer according to histological grade could be decoded. To this end, a LOOC SVM strategy was applied based on unfiltered microarray data. The results showed that grade 1 vs. grade 3 tumors could be classified, with a ROC AUC value of 0.83 (Fig 1A). In contrast, grade 2 tumors could not readily be differentiated from neither grade 1 nor grade 3 tumors, as illustrated by ROC AUC values of 0.67 and 0.59, respectively (Fig 1B and 1C). Hence, the data indicated on a large molecular heterogeneity among the grade 2 tumors. Visualizing the microarray data using a PCA based approach (Fig 1), a similar pattern of discrimination was observed, further supporting the conclusions.

A total of 170 proteins were found to be significantly differentially expressed (p < 0.05) between grade 1 vs. grade 3 tumors, further highlighting the distinct differences between these two grades (Table 3; top 20 differentially expressed proteins listed). Among the top 20 proteins, all (*e*.*g*. IL-6, Angiomotin, MCP-1 and CDK-2) but Mucine-1, were found to be present at higher levels in grade 3 than in grade 1 tumors. In this context, it might be interesting to note that several antibody clones targeting the same protein (*e*.*g*. four anti-IL-6 antibodies), but directed against different epitopes, gave similar binding patterns, further supporting the observations. In contrast, the number of significantly differentially expressed (p < 0.05) proteins was only found to be 23 and 19 for grade 2 vs. grade 1 or grade 3, respectively (Tables 4 and 5), further outlining the small differences between these grades. In the case of grade 2 vs. grade 1, all proteins (*e*.*g*. IL-2, IL-3, IL-4, IL-5, IL-6, Lewis y, CD40 ligand, and Angiomotin), but two (Mucine-1 and Cystatin C), were found to be present at higher levels in grade 2 than in grade 1 tumors. For grade 2 vs. grade 3 tumors, all proteins (*e*.*g*. IL-6, IL-8, CDK-2, UBE2C, and UPF3B), but three (Factor B, C3, and CIMS-29), were found to be present at higher levels in grade 3. Taken together, the data showed that several candidate protein biomarkers associated with histological grade of breast cancer could be delineated.

**Table 3:** Significant analytes from SVM leave one out cross validation on unfiltered data for H1 vs. H3.

Protein Names	Foldchange	Wilcoxon p-values	QvaluesAll
Angiomotin (2)	6.59E-18	0.0003	0.027
CD40 ligand (1)	1.99E-13	0.0005	0.027
IL-6 (2)	3.78E-14	0.0005	0.027
Leptin (1)	2.59E-13	0.0007	0.027
IL-6 (5)	7.12E-08	0.0007	0.027
IL-6 (3)	1.12E-13	0.0007	0.027
Her2/ErbB2 (1)	1.57E-15	0.0009	0.027
IL-1ra (1)	1.40E-13	0.0009	0.027
CHX10 (2)	4.56E-14	0.0011	0.027
MUC1 (3)	7.99E+09	0.0011	0.027
Sox11a (1)	2.69E-13	0.0013	0.027
MCP-1 (8)	1.34E-12	0.0013	0.027
MCP-3 (1)	7.78E-11	0.0013	0.027
MCP-3 (2)	3.81E-19	0.0013	0.027
PSA (1)	8.50E-10	0.0015	0.027
CDK-2 (2)	7.64E-05	0.0018	0.027
Apo-A4 (2)	2.66E-12	0.0018	0.027
ORP-3 (1)	5.42E-10	0.0018	0.027
GM-CSF (1)	7.84E-12	0.0018	0.027
IL-3 (3)	3.54E-11	0.0018	0.027
IFN-γ (2)	4.31E-09	0.0022	0.027
IL-4 (2)	4.67E-13	0.0022	0.027
FASN (1)	1.65E-13	0.0026	0.027
Her2/ErbB2 (2)	1.49E-09	0.0026	0.027
Apo-A4 (1)	1.46E-12	0.0026	0.027
GM-CSF (2)	3.26E-12	0.0026	0.027
IL-6 (1)	7.32E-11	0.0026	0.027
TGF-b1 (3)	4.53E-11	0.0026	0.027

**Table 4:** Significant analytes from SVM leave one out cross validation on unfiltered data for H1 vs. H2.

Protein Names	Foldchange	Wilcoxon p-values	QvaluesAll
Lewis y	8.78E-08	0.002	0.406
CIMS (13)	3.24E-16	0.011	0.406
MUC1 (3)	3.00E+07	0.013	0.406
MUC1 (4)	1.39E+08	0.013	0.406
IL-2 (3)	8.71E-11	0.016	0.406
IL-3 (3)	6.58E-09	0.021	0.406
IL-5 (2)	2.12E-09	0.021	0.406
CD40 ligand (1)	1.45E-10	0.025	0.406
Cystatin C (3)	2.50E+11	0.025	0.406
MUC1 (6)	1.39E+08	0.025	0.406
Angiomotin (2)	3.11E-15	0.029	0.406
IL-6 (5)	7.39E-05	0.029	0.406
Cystatin C (4)	4.44E+09	0.034	0.406
Sox11a (1)	2.26E-09	0.034	0.406
MCP-3 (1)	1.14E-07	0.034	0.406
C5 (2)	2.55E-08	0.039	0.406
C5 (3)	1.35E-10	0.039	0.406
CD40 (1)	1.07E-10	0.039	0.406
IL-2 (2)	8.15E-10	0.039	0.406
Leptin (1)	1.16E-10	0.045	0.406
CIMS (14)	3.44E-10	0.045	0.406
IL-4 (4)	2.65E-08	0.045	0.406
IL-5 (1)	9.85E-09	0.045	0.406

**Table 5:** Significant analytes from SVM leave one out cross validation on unfiltered data for H2 vs. H3.

Protein Names	Foldchange	Wilcoxon p-values	QvaluesAll
Factor B (4)	5.46E+07	0.0004	0.121
CIMS (29)	5.22E+06	0.007	0.517
Factor B (2)	8.40E+05	0.013	0.517
Osteopontin (2)	1.95E-07	0.014	0.517
IL-8 (2)	5.84E-08	0.020	0.517
IL-6 (2)	2.92E-07	0.024	0.517
CIMS (5)	2.90E-07	0.027	0.517
UPF3B (2)	2.31E-04	0.027	0.517
CDK-2 (2)	1.04E-03	0.032	0.517
IL-6 (3)	3.80E-07	0.032	0.517
CDK-2 (1)	7.07E-04	0.034	0.517
UBE2C (1)	7.73E-24	0.037	0.517
Integrin α10 (1)	2.97E-07	0.039	0.517
IgM (1)	1.87E-03	0.039	0.517
C3 (4)	6.95E+03	0.042	0.517
Factor B (1)	1.11E+05	0.045	0.517
IL-10 (2)	9.51E-07	0.045	0.517
MCP-4 (1)	6.56E-06	0.045	0.517
Apo-A4 (2)	6.39E-05	0.048	0.517

### Validation of protein expression profiles

**Fig 2:**
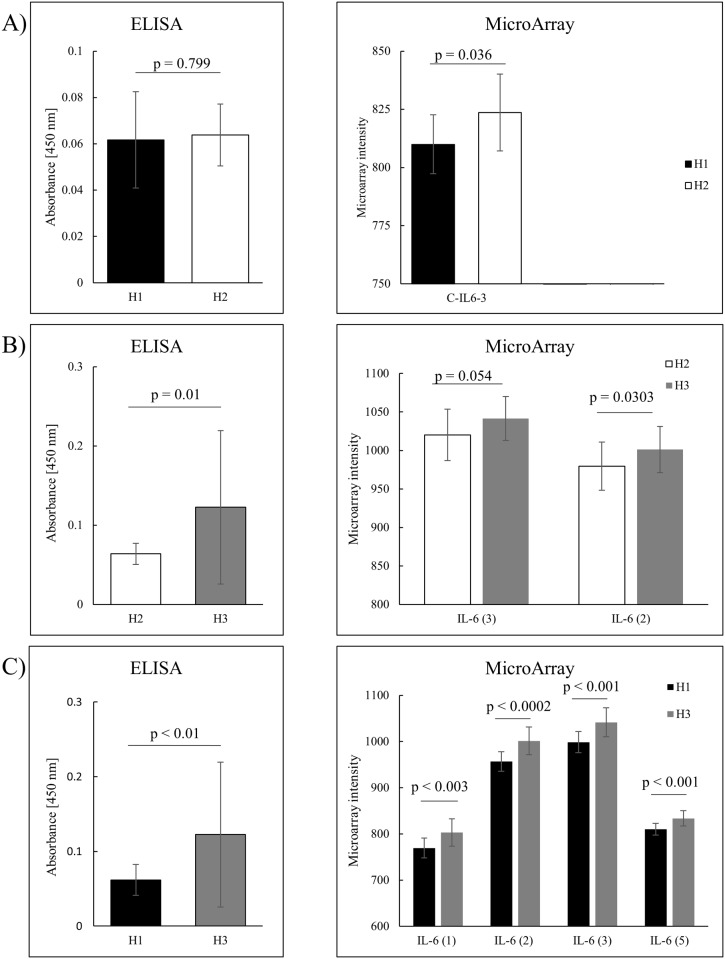
Validation of antibody microarray data using an orthogonal method (ELISA). A) Histological grade 1 vs. 2 based on ELISA data (left panel) and antibody microarray data (right panel). B) Histological grade 2 vs. 3, based on ELISA data (left panel) and antibody microarray data (right panel). C) Histological grade 1 vs. 3, based on ELISA data (left panel) and antibody microarray data (right panel). In all comparisons, a Welsh t-test was used to evaluate the level of significance.

In an attempt to validate the recombinant antibody microarray data, the observed protein expression profiles were compared with those obtained using an orthogonal, commercially available method (ELISA) (Fig 2). To this end, IL-6 was selected as model protein, since i) it was indicated to be significantly differentially expressed in all LOOC SVM analysis, and ii) several antibodies targeting IL-6, but directed against different epitopes, were included on the arrays. The results showed that the differential expression pattern of IL-6 observed using microarrays was reproduced using the ELISA method (Fig 2). Hence, the microarray data was validated by an orthogonal, independent method.

### First model for refined molecular grading of breast cancer

**Fig 3:**
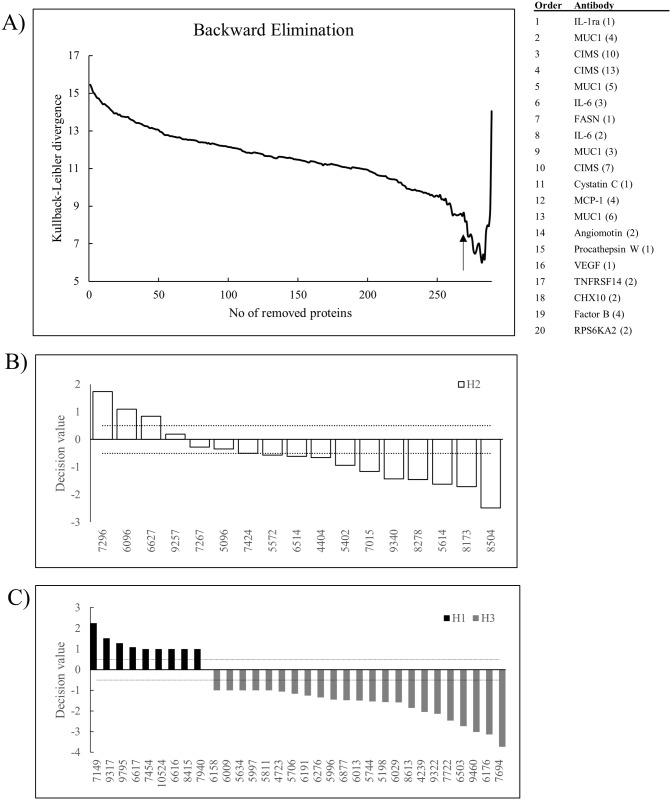
First model for refined molecular grading of breast cancer. A) Backward elimination analysis of the data set (grade 1 and grade 3 tumors), resulting in a condensed signature of 20 antibodies (indicated by an arrow). The panel of antibodies (specificities) are shown (in order of last removed antibody). B) A frozen SVM classification model was generated using the 20-plex antibody panel in A, based on all grade 1 and 3 tumors. The grade 2 tumors were then applied as test set. The resulting classification decision values are shown, where tumors with values ≥ 0.5 are defined as being more similar grade 1 tumors, 0.5 to -0.5 is defined as a grey zone (i.e. grade 2 tumors), and ≤ -0.5 are defined as being more similar to grade 3 tumors. C) The decision values for the grade 1 and grade 3 tumors used to build the SVM model are plotted. The same arbitrary cut-off as in B) is indicated (dashed line).

To explore whether a model for refined molecular grading of breast cancer, and in particular grade 2 tumors, could be generated, we used the grade 1 and 3 tumors (the best defined grades) to build a classification model. In order to define a condensed list with those protein biomarkers that contributed the most to the classification of grade 1 vs. 3 (as opposed to the list of proteins based on p-values, merely indicating whether they were significantly differentially expressed (Table 3), a backward elimination strategy was adopted (Fig 3). The top 20 proteins (*e*.*g*. antibodies) most important for classifying grade 1 vs. grade 3 tumors are shown in Fig 3A, including e.g. IL-6, Mucine-1, Cystatin C, and Angiomotin. Of note, among the identified candidate biomarker proteins, four proteins were pin-pointed (VEGF, Procathepsin W, TNFRSF14, and PS6KA2), which would have been missed if the signature had been selected based simply on p-values.

The grade 1 and 3 tumors were then used to calibrate the SVM model using this 20 protein signature, where after the grade 2 tumors were classified using the frozen model (Fig 3B). A nominal cut-off, or grey zone, was adopted, defined by a decision value set to ± 0.5. While four tumors were still considered to be of grade 2, three were re-classified as being more similar to grade 1, and ten re-classified as being more similar to grade 3. Hence, the data, implied that molecular profiling could be used for refined molecular grading of breast cancer, in particular of the heterogeneous grade 2 tumors.

Testing the model on the grade 1 and 3 tumors resulted in a 100% correct classification (Fig 3C). This comparison is, of course, biased, as all these samples were used to build the model. The current model could potentially also be limited by the fact that the á priori determined grading of the tumors used to build the model was assumed to be correct. Since, this criteria might not be a 100% correct, a new model at least to some extent bypassing this issue should be designed.

### Improved models for refined molecular grading of breast cancer

**Fig 4:**
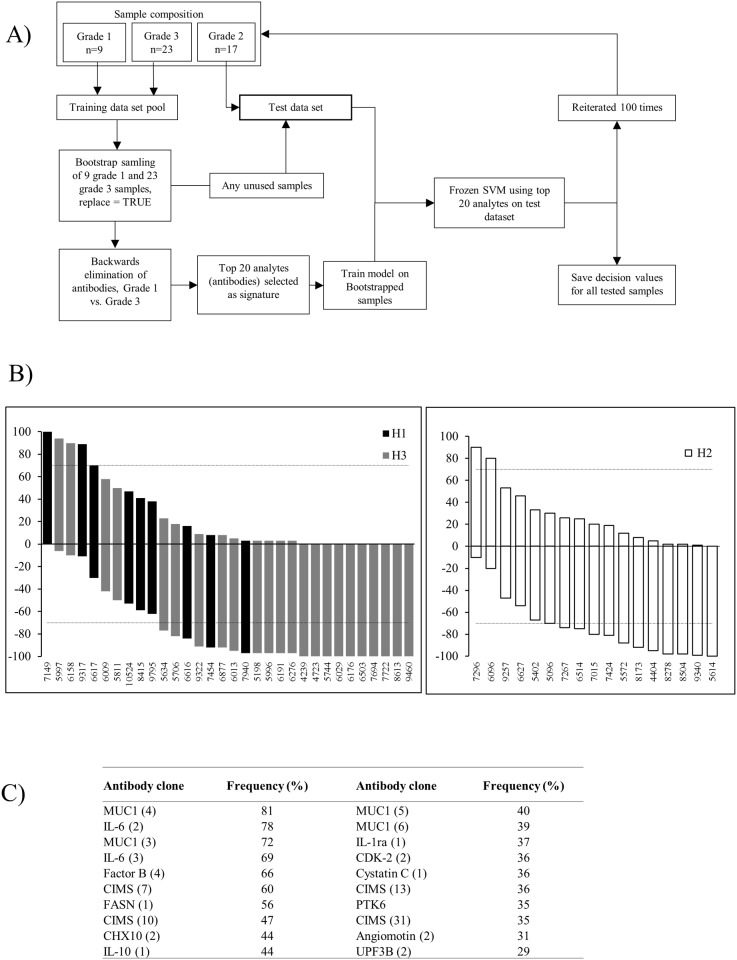
Second model for refined molecular grading of breast cancer. A) Outline of the sample bootstrap strategy approach, combined with 100 iterative cycles of backward elimination and frozen SVM, generating a classification model. B) The classification is shown in terms of number of time a tumor was classified as either grade 1 (positive value) or grade 3 (negative value). A tumor was classified as being more similar to grade 1 when the value was ≥70, <70 to >-70 is defined as a grey zone (i.e. grade 2 tumors), and ≤ -70 are defined as being more similar to grade 3 tumors. The arbitrary cut-limits are indicated by dashed lines. Left panel–classification of grade 1 and grade 3 tumors. Right panel–classification of grade 2 tumors. C) Consensus list of the twenty most often occurring antibody clones in the condensed signatures.

In order to build a more adequate model for molecular grading of breast cancer, a (sample) bootstrap strategy was implemented (Fig 4A). By randomly picking samples from grade 1 and 3 tumors with re-sampling, respectively, a training dataset was generated. Next, a condensed 20-plex protein signature was generated using the backward elimination strategy, and used to train a frozen SVM model. Any unused grade 1 and grade 3 tumors were then added to the test dataset (previously only composed of grade 2 tumors) and independently tested using the frozen model (the SVM decision values were saved). This entire procedure was repeated 100 times. Based on the number of times a tumor was classed as grade 1 or 3, a nominal cut-off at 70% was adopted to classify tumors as grade 1, 2 or 3.

The analysis showed that two grade 2 tumors were re-classified as being more similar to grade 1, four tumors remained in the grey zone as grade 2 tumors, while eleven tumors were re-classified as being more similar to grade 3 (Fig 4B). Compared to the first model, the classification overlapped for 14 of 17 samples (cfs. Figs 3B and 4B). Of note, the refined model also indicated that the grade 1 and 3 tumors were no longer classified as a 100% correct compared to the á priori grading, indicating a tentative need for re-classification here as well for several tumors (Fig 4B).

However, the adopted model strategy was based on generating a unique 20-plex protein signature for each iterative round. This means that the signature will differ from round to round, reflecting the precise composition of the training dataset (data not shown). Depending on how the samples were selected (*e*.*g*. whether the most typical/atypical tumors samples were included/excluded) will thus have a significant impact, in particular when targeting relatively small sample cohorts. In Fig 4C, the top 20 most frequently included in the signature are shown. The frequency ranged from high (>60%; *e*.*g*. IL-6, Mucine-1, and Factor B) to low (<40%; *e*.*g*. CDK-2 and Angiomotin), illustrating the influence of sample selection. Instead of using 100 different signatures, adopting this list of biomarkers as a consensus signature could be a way to bypass, or minimize, the observed sample dependency.

**Fig 5:**
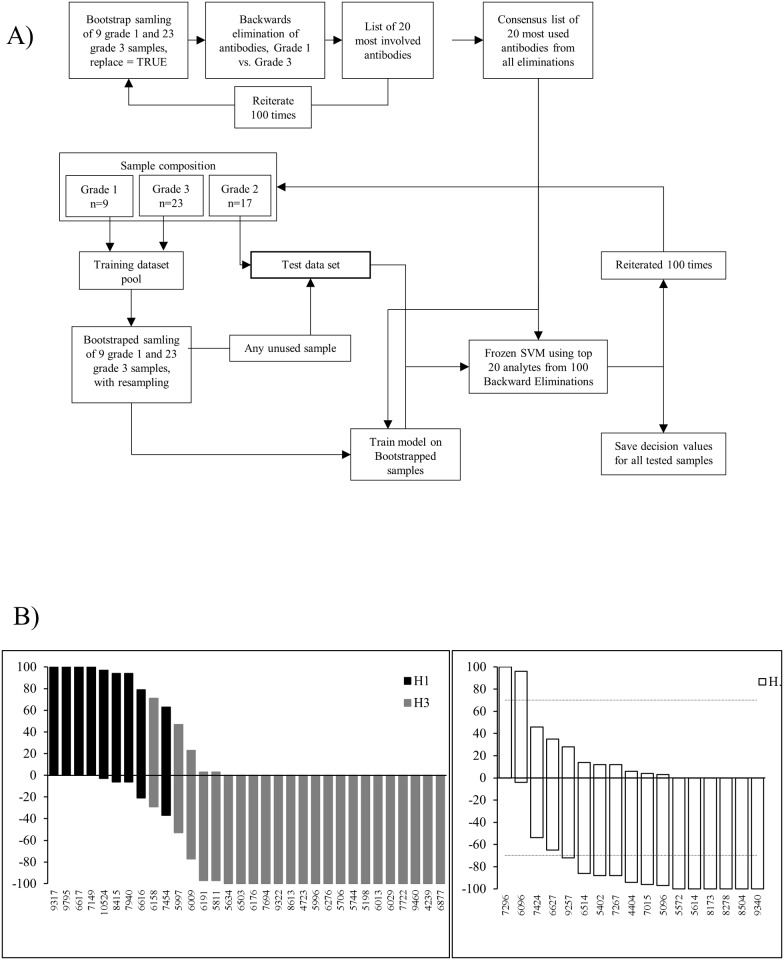
Third model for refined molecular grading of breast cancer. A) Outline of the sample bootstrap strategy and combination with backward elimination and frozen SVM, generating a classification model. B) The classification is shown in terms of number of time a tumor was classified as either grade 1 (positive value) or grade 3 (negative value). A tumor was classified as being more similar to grade 1 when the value was ≥70, <70 to >-70 is defined as a grey zone (i.e. grade 2 tumors), and ≤ -70 are defined as being more similar to grade 3 tumors. The arbitrary cut-limits are indicated by dashed lines. Left panel–classification of grade 1 and grade 3 tumors. Right panel–classification of grade 2 tumors.

Using the consensus signature as a fixed protein signature (Fig 4C), the bootstrap strategy with re-sampling was re-run and a new classification model was generated (Fig 5A). The analysis showed that two grade 2 tumors were re-classified as being more similar to grade 1, three tumors remained in the grey zone as grade 2 tumors, while twelve tumors were re-classified as being more similar to grade 3 (Fig 5B). Further, the data (model) also indicated that one grade 1 tumor and two grade 3 tumors ended up in the grey zone (*i*.*e*. as grade 2 tumors), while the remaining tumors were apparently classified in accordance to the á priori grading.

Taken together, this pilot study showed that new candidate biomarker signatures reflecting histological grade of breast cancer, and in particular the heterogeneity of grade 2 tumors, could be pin-pointed targeting the immunoproteome. This will add additional information to the underlying molecular puzzle of (grading) breast cancer tumors.

## Discussion

Finding protein biomarkers for accurate grading and molecular classification of breast cancer tumors is of great clinical relevance, by which the patients in the end could benefit by tailored care. Clinical immunoproteomics is based on exploring the immune system as a disease-specific sensor [29]. We have previously demonstrated the ability of our recombinant antibody microarray technology platform [28, 46], targeting mainly immunoregulatory proteins, as a unique tool for serum biomarker discovery within cancer [28, 47], including breast cancer [41, 42]. In the latter case, tentative serum biomarker signatures reflecting metastatic breast cancer [42] and predicting the development of distant metastases [41] were discovered. In this pilot study, we have for the first time explored the immunoproteome of breast cancer tumor tissue for disease-associated protein biomarkers reflecting histological grade, using our antibody array technology platform. Uniquely, the results showed that several tissue proteins reflecting histological grade could be delineated. In more detail, the candidate biomarker signatures could be used to i) build a tentative model classifying grade 1 vs. grade 3 tumors, ii) demonstrate the molecular heterogeneity among grade 2 tumors, and iii) potentially re-classify several of the grade 2 tumors to more like grade 1 or grade 3 tumors. Hence, these new molecular features supported the notion presented by others that grade 2 tumors represents a heterogeneous cohort that could benefit from being re-classified [8, 9, 12, 22]. Follow-up studies, based on larger, independent sample sets, will be required to (pre-)validate these findings and to examine whether this new classification of grade 2 tumors was better correlated with e.g. survival.

Of note, these key observations were also (indirectly) supported by our recent profiling efforts, where we analysed the same tissue samples, but i) explored a completely different part of the proteome, and ii) and used different technologies, either a mass-spectrometry based affinity approach [24] or a targeted mass spectrometry method (Olsson et al, submitted). This work indicated a candidate 49-plex protein signature [24], refined and extended to a tentative 21-plex peptide signature (Olsson et al, submitted), for discriminating between histological grade 1,2 and 3 classified breast cancer, and for outlining the heterogeneity among grade 2 tumors. Importantly, these candidate biomarker signatures are completely different from the biomarker panels presented in this study, but they were found to provide similar biological information. Hence, this illustrated the importance of the experimental design for the end result. But more interestingly, it demonstrated the massive amount of key biological information (in terms of biomarker panels) potentially carried by the proteome just waiting to be harvested. Additional work will be required to explore the power of combining these tentative signatures and/or for exploring the remaining part of the proteome for additional relevant information reflecting histological grade of breast cancer tumors.

The observed differences of immunophenotype between grade 1 and grade 3 tumors did not merely reflect differences in receptor status (only triple negative in grade 3 tumors, 8 of 23) (data not shown). Briefly, the classification of grade 1 vs. grade 3 tumors gave similar AUC values (0.83 vs. 0.85) whether all grade 3 tumors were included or the triple negative tumors were excluded (n = 23, of which 8 were triple negative). When comparing the top 30 differentially expressed proteins for these two classifications, 21 of 30 biomarkers overlapped. Noteworthy, only minor differences could be observed, more reflecting the receptor status, as indicated when comparing only grade 3 tumors, divided into two groups—triple negative (n = 8) vs. positive (n = 15) tumors. In addition, the list of top 30 differentially expressed proteins did not correlate with the above lists (only 1 of 30 overlapped), further indicating that the observed differences of immunophenotype between grade 1 and grade 3 tumors did not only mirror differences in receptor status. Additional studies will be needed to validate also these findings.

This pilot study does have some limitations, such as the number of tumor samples analysed and the number and range of antibody specificities included. In this context, it should be noted that we, where possible, adopted stringent biostatistical methods, such as leave-one out cross validation and (sample) bootstrap strategies, as one way of addressing the issue of limited samples. Still, the data and SVM models needs to be corroborated in follow-up studies targeting larger, independent patient cohorts collected at different sites. Regarding the density of the antibody arrays, we used a 293-plex recombinant antibody microarray, of which 262 were targeted against 98 known proteins, and 31 were directed against short peptide motifs. But despite the fact that our set-up only targeted a selected part of the immunoproteome, we were still capable of deciphering several disease-associated and discriminatory protein biomarkers. It could thus be rewarding the further explore the remaining part of the immunoproteome in future experiments.

However, it should be noted that several of the biomarkers that we identified have previously been indicated with breast cancer (and histological grade thereof), but not in terms of multiplex signatures as in our case. This illustrates the uniqueness of our approach and supports and highlights the biological relevance of our observations. A majority of all significantly differentially expressed proteins were found to be present at higher levels with increasing histological grade, *i*.*e*. in the order of grade 1< grade 2 < grade 3.

Mucin-1 was one of the few proteins present at higher levels at lover grade. Although debated, studies have suggested increased expression of Mucin-1 at lower grade as a prognostic value [48], which would support our observations. More commonly, shredded or soluble forms of Mucin-1 [49, 50] are measured as a serological clinical marker for monitoring response to treatment in breast cancer [51].

Among the proteins present at higher level at higher grade, IL-6 was frequently pinpointed. IL-6 is a pleiotropic cytokine, which acts directly on cancer cells to promote their survival and proliferation [52]. Elevated serum levels of IL-6 have also been shown to negatively correlate survival of cancer patients, which might be attributed to defective responses of patients T cells to IL-6 [53]. In addition, IL-6 has been reported to be involved, with other chemokines (*e*.*g*. IL-2, IL-3, IL-4, and IL-5), in key mechanisms of tumorigenesis, and as a factor in certain EMT pathways that contributes to metastatic processes [54]. Notably, the levels of IL-2, IL-3, IL-4, and IL-5 were also found to be higher in grade 3 vs. grade 1 tumors, further supporting our observations.

Furthermore, CD40 and CD40L were also found to be among the proteins with higher levels in grade 3 vs. grade 1. In accordance, the expression levels of CD40/CD40L have also been found to be increased in breast cancer, displaying a positive relationship with pathological grade [55]. A recent study, showed that the production of TGF-β induced by the CD40-CD40L interaction resulted in enhanced immunosuppressive function of breast cancer cells, thereby contributing to tumor progression [55].

Another protein displaying increased levels with grade was Cyclin Dependent Kinase 2 (CDK-2). CDK-2 is a serinine/threonine kinase involved in the control of cell cycle, and phosphorylates among others, p53 and BRCA2 [56, 57]. Notably, phosphorylated CDK-2 has been indicated as a biomarker for aggressive breast cancer [58].

Cystatin C, Fatty Acid Synthase (FASN), Complement factor B, IL-1ra, PTK6, and Angiomotin were some additional proteins found to be important in the condensed biomarker signatures. Cystatin C, a major inhibitor of Cathepsins, has been identified as a novel p53 target, and the levels of Cystatin C was found to be associated with poor prognosis of breast cancer [59]. Previous studies have shown a strong correlation between FASN and the aggressiveness of breast cancer, and increased levels are associated with poor prognosis [60, 61]. The levels of complement factor B has been shown to be increased in breast cancer [62] and associated with molecular subtypes of breast cancer [63]. IL-1 has been shown to act as a tumor suppressor, and the IL-1 receptor antagonist (IL-1ra) binds the receptor of IL-1 without activating it. Elevated levels of IL-1ra has been linked to tumor load [64, 65]. Further, PTK6 has been indicated as a prognostic factor for long-term breast cancer survival [66]. Finally, Angiomotin is a multifunctional protein involved in endothelial cell migration and tube formation and angiogenesis. Recent data has shown Angiomotin to be highly expressed in breast cancer tissue and to be important for promoting breast cancer cell proliferation and invasion [67]. Taken together, the biological and clinical relevance of the breast cancer associated protein biomarkers deciphered in this study was thus strongly supported by all of these studies. From a biological point of view, the data could be interpreted as that the grade 3 tumors have shaped their (micro)environment in a way to increase the survival and metastatic ability of tumors cells compared to grade 1 tumors.

In conclusion, we have in this pilot study shown that the immunoprotome of breast cancer tissue contained key biological information in terms of protein biomarkers that could be deciphered using recombinant antibody microarrays. More specifically, candidate biomarker signatures differentiating grade 1, 2 and 3 tumors as well outlining the molecular heterogeneity among grade 2 tumors were delineated. The study have thus provided additional key information about the underlying molecular puzzle of breast cancer and histological grade thereof. This could, once validated and further refined, improve the prognostic ability, in the end resulting in improved tailored patient care (*e*.*g*. improved decision in therapy selection).

## Supporting information

S1 Table Raw antibody microarray data. The observed array signal intensity for each antibody is listed per sample.
(XLSX)

## Abbreviations

AUC: area under the curve
KL: Kullback-Leibler error
ROC: receiver operating characteristics
scFv: single-chain fragment variable
SVM: support vector machine
